# Empirical development of a typology on residential long-term care units in Germany - results of an exploratory multivariate data analysis

**DOI:** 10.1186/s12913-020-05401-4

**Published:** 2020-07-11

**Authors:** Johannes Michael Bergmann, Armin Michael Ströbel, Bernhard Holle, Rebecca Palm

**Affiliations:** 1 German Centre for Neurodegenerative Diseases (DZNE), Stockumer Str. 12, Witten, North Rhine-Westphalia 58453 Germany; 2 University Witten/Herdecke, Faculty of Health, Department for Nursing Science, Witten, North Rhine-Westphalia Germany; 3 Center for Clinical Studies, University Hospital Erlangen, Krankenhausstraße 12, Erlangen, Bavaria 91054 Germany

**Keywords:** Nursing, Multiple correspondence analysis, Explorative, Typology, Care structures, Residential long-term care, Hierarchical clustering

## Abstract

**Background:**

Organizational health care research focuses on describing structures and processes in organizations and investigating their impact on the quality of health care. In the setting of residential long-term care, this effort includes the examination and description of structural differences among the organizations (e.g., nursing homes). The objective of the analysis is to develop an empirical typology of living units in nursing homes that differ in their structural characteristics.

**Methods:**

Data from the DemenzMonitor Study were used. The DemenzMonitor is an observational study carried out in a convenience sample of 103 living units in 51 nursing homes spread over 11 German federal states. Characteristics of living units were measured by 19 variables related to staffing, work organization, building characteristics and meal preparation. Multiple correspondence analysis (MCA) and agglomerative hierarchical cluster analysis (AHC) are suitable to create a typology of living units. Both methods are multivariate and explorative. We present a comparison with a previous typology (created by a nonexplorative and nonmultivariate process) of the living units derived from the same data set.

**Results:**

The MCA revealed differences among the living units, which are defined in particular by the size of the living unit (number of beds), the additional qualifications of the head nurse, the living concept and the presence of additional financing through a separate benefit agreement. We identified three types of living units; these clusters occur significantly with a certain combination of characteristics. In terms of content, the three clusters can be defined as: “house community”, “dementia special care units” and “usual care”.

**Conclusion:**

A typology is useful to gain a deeper understanding of the differences in the care structures of residential long-term care organizations. In addition, the study provides a practical recommendation on how to apply the results, enabling living units to be assigned to a certain type. The typology can be used as a reference for definitions.

## Background

In Germany, nursing homes are an important part of health care organizations. At present, more than 11,000 institutions are providing service for more than 800,000 people [1]. Nursing homes vary enormously with regard to their structural characteristics.

Nursing homes may be affiliated with owners who have different business objectives (for-profit vs. nonprofit); they can be organized as chains with superordinate policies and regulations; they can provide more than 300 beds or fewer than 10, and they may be organized in separable units with different teams and philosophies of care. Additionally, their service mission is multilayered: they deliver professional nursing care, provide opportunities for social interaction and participation for their residents, and ensure that medical care by general physicians and specialists is being delivered and prescribed therapy is received. Likewise, nursing homes are expected to provide an environment that maintains their residents’ preserved skills and that supports people with dementia in acting and making decisions autonomously for as long as possible. Nursing homes are also expected to provide an environment in which residents feel at home – not institutionalized – and can thus maintain their quality of life on the highest possible level [2].

German nursing home residents all share the attribute of being approved as care-dependent by the Long Term Care Insurance entity, which enables them to receive benefits. Because this financial system especially supports people who want to stay at home as long as possible, they do not move to a nursing home until their need for care exceeds what can be provided at home. As a result, nursing home residents are predominantly severely care-dependent; more than 70% are affected by the consequences of a dementia [3, 4].

In recent years, as problems with health care outcome quality have become public, the quality of care in nursing homes has attracted more political and scientific attention. In particular, it was reported that the needs of people with dementia were not being sufficiently addressed [5]. As a result, nursing homes implemented various approaches to dementia care that necessitated some changes in organizational structure. One major change was to use designated care units to separate residents with dementia from residents without cognitive impairments, under the assumption that care for residents with dementia could be better provided in a special environment. The implementation of “Dementia Special Care Units” (DSCUs) was a worldwide development that had its origin in the United States. In Germany, it is estimated that 30–50% of nursing homes have implemented at least one DSCU [5, 6]. Internationally, many different models for nursing homes have been developed [7]. Examples are the Green House model in the USA (Kane et al., 2007), small scale living facilities in the Netherlands [8], and the Besondere stationäre Dementenbetreuung in Germany [9]. They are characterized by an adapted physical environment that supports homelikeness and avoids an institutional character; adapted staff roles that enhance person-centered attitudes, beliefs and values and avoid paternalism; and shifted tasks that prioritize meaningful activities stronger than cure and care routines. The question of whether DSCUs provide better outcomes has been the subject of large research projects throughout the world. Leading researchers from the United States concluded after 20 years of research that *“(D)SCUs have been effective in changing certain processes of care that are associated with positive behaviors among dementia residents, the impact of such changes […] on cognitive or functional performance appear negligible. In fact, (D)SCUs […] may have the most demonstrable benefits for cognitively intact residents, their families and nursing home staff* ”[7]. The authors of a Cochrane Review concluded*, “There is limited evidence to support the assumption that the care of people with dementia in special care units is superior to care in traditional care units. It is probably more important to implement best practice than to provide a specialized care environment.* ”[10]. Additionally, the latest reviews on the question of whether DSCUs provide a better quality of care and whether residents experience better outcomes do not show consistent results in favor of DSCU models [11]. The role that facility characteristics play in explaining resident outcomes remains unclear [12, 13].

One reason why evaluation studies of DSCUs failed to produce explicit results may be that the interpretation of existing quasi-experimental studies is complicated because they are prone to many sources of nonrandom error [14]. One described challenge for the interpretation of the study results is the lack of definitional clarity for DSCUs [15]. Whereas in the U.S., DSCU typologies have been developed in response to this lack of clarity [16, 17], such typologies are still missing in Germany.

We conducted a study in German nursing homes that aimed to identify resident- and facility-related factors that are associated with the nursing home residents’ health care outcomes (DemenzMonitor study) [18]. The longitudinal study DemenzMonitor was conducted in the period from 2011 to 2014. One goal was to answer the question of whether we can find differences in the quality of care and in residents’ outcomes between living units that are dementia-specific and traditional care units. For reach this purpose, the initial aim was to investigate differences in the structural characteristics of different types of living units in German nursing homes based on the characteristics that are usually used to define dementia-specific care units (size, segregation of residents with dementia, extra funding for additional staff resources) [2, 11]. Next, we wanted to know if we would find differences between living units that are small and large because it is proposed that small living units are beneficial for people with dementia (ibid). Furthermore, we assumed that the extra funding some of the units received to finance more staff was also an important definition criterion. Based on these structural characteristics, eight types of different living units could have been defined a priori, but only these five types could be realized in the data. Therefore, in the absence of a typology for DSCUs and traditional care units, we defined the following types of living units [19]:

1. Large segregated living units without extra funding (LSLU I)
2. Large segregated living units with extra funding (LSLU II)
3. Large integrated living units without extra funding (LILU)
4. Small segregated living units without extra funding (SSLU)
5. Small integrated living units without extra funding (SILU)

Observing these five types, we expected that large segregated living units with extra funding (LSLU II) would have better staff resources, accommodate residents with more severe symptoms of dementia, provide a milieu that is more dementia-friendly and perform better with respect to national guidelines in the care of people with dementia and challenging behavior in comparison with the other types of living units. Hence, we also expected to find differences among the living units with respect to the residents’ care outcomes. In fact, the results of our subsequent analysis confirmed these assumptions only partly. Finally, we could not show that residents of dementia-specific (segregated) living units or small living units had better care outcomes compared to residents from other living units [20]. One reason why we did not find the expected results may be the a priori definition of the living unit types. This may be because the structural characteristics chosen for the typology do not adequately describe the complex differences between the living units.

In our study protocol of the DemenzMonitor study, we additionally formulated the aim of investigating types other than dementia-special/traditional/large/small living units. On this basis, we have included many additional characteristics for type formation in the current study. To achieve this, we have oriented toward our previous results, because they indicate that the types defined a priori are associated with a variety of other structural characteristics [19], implying that there are more complex relationships that can be considered for the development of living unit types. These are structural characteristics of the living units, which are defined by variables relating to staffing, such as “Special qualification of head nurse in psychogeriatric care”, and by variables relating to spatial conditions, such as “Architectural segregation from other units”. Thus, although these previous results already contained helpful empirical information on the formation of such types, there is no detailed knowledge on how all these structural characteristics interact and what contribution they make to a more complex type of formation. Because we observed more than 30 relevant structural characteristics for living units in the DemenzMonitor study, we decided to pursue our objective by conducting a multivariate analysis. For this reason, the aim of the present study is to develop an empirical typology of living units based on all these structural characteristics in order to systematically map differences among them. Instead of using a priori defined structural characteristics to define different types, we will use an explorative clustering technique that identifies the characteristics that are most relevant. The difference from the previous analysis is that with this approach, the numbers and types of clusters are calculated by a “data-driven” analysis. We aim to compare the results of the typology with our previous published results on the structural characteristics of the living unit types to conclude whether the applied methods are meaningful with respect to the typology development of the living units.

The present article will provide answers to the following research questions:

1. How many clusters of care units with similar characteristics can be identified?
2. Which characteristics are most important when identifying clusters (because they contribute the most to the cluster structure)?
3. To what extent do the identified clusters differ from those previously published?

## Methods

### Design and sample

For the study, cross-sectional data from a convenience sample of 103 living units in 51 nursing homes were used. The data are from the 2013 measurement period of the DemenzMonitor study [18]. This is the same data source on which the previous definition of types was performed, which allows a direct comparison of the results. Participating nursing homes were defined according to the German statutory long-term care insurance law, under which people in need of care are reimbursed by the statutory long-term care insurance.

Beyond that, there were no inclusion or exclusion criteria for the participation of nursing homes; diversity was intended. The nursing homes that declared their interest in participating were included. All nursing homes participated voluntarily.

### Data collection

The data were collected by the nursing home staff using a standardized questionnaire. Therefore, specific questionnaires were developed and tested. The details of the questionnaire development are described in depth elsewhere [18, 19].

The data were collected with paper-pencil questionnaires or by directly entering them into an online database. Questionnaires were filled out by the nursing home staff. The paper questionnaires were sent out to the participating nursing homes by post and returned to the German Centre for Neurodegenerative Diseases. Data entry into SPSS was conducted by a professional agency. Nursing homes that collected the data online received a link to the data platform. These data were collected in separate questionnaires at the level of the nursing home, the living units and the residents. The living unit questionnaire was completed by the head nurse; the nursing home questionnaire was completed by the nursing home manager; and the resident questionnaire was completed by a registered nurse familiar with the resident. More details on data collection can also be obtained from previous reports [6, 19].

### Variables and measurements

For the present study, we evaluated the same variables for the structural characteristics of the living units that were used in the previously published results [19]. These are variables for structural characteristics such as the organization of meal services, size of the living unit, interior design, architectural characteristics, staffing, etc. The data level of the variables is exclusively categorical. An overview of the variables and their measurement is provided in Table 1.

**Table 1:** Overview of variables and their measurement

Variable	Categories	Shortname
Size of the living unit	Number of beds in living unit ≤15	Size 0
	Number of beds in living unit > 15	Size 1
Availability of single rooms	Living units do not exclusively have single rooms.	SRoom 0
	Living units have only single rooms.	SRoom 1
Building specific for residents with dementia	The living unit was not specially built for people with dementia.	Build 0
	The living unit was built specially for people with dementia.	Build 1
Architectural segregation from other units	The living unit is not located in a separate building or floor and is not separated by a closed door.	Separate 0
	The living unit is located in a separate building, floor or is separated by a closed door.	Separate 1
Exit control	The living unit is not protected by an exit control.	Guarded 0
	The living unit is protected by exit controls.	Guarded 1
Furnishing of public rooms	Furnishings are solely functional (Functional furniture is provided by the institution and designed for a special use.)	Furniture 0
	Furnishings are functional and individual (Individual furniture is purchased from private individuals.)	Furniture 1
Opportunities to cook lunch in the living unit	Lunch is not cooked in the kitchen of the living unit.	Selfcook 0
	Lunch is cooked in the kitchen of the living unit.	Selfcook 1
Meal serving system	All meals (breakfast, lunch and dinner) are not served homestyle on the table (tray system, dish system, buffet system or mixed system).	Mealserv 0
	All meals (breakfast, lunch and dinner) are served homestyle on the table.	Mealserv 1
Constant assignment of nurses	Nurses do not work exclusively in one designated living unit.	AssignN 0
	Nurses work exclusively in one designated living unit.	AssignN 1
Constant assignment of service staff	Service workers do not work exclusively in one designated living unit.	AssignSSM 0
	Service workers work exclusively in one designated living unit.	AssignSSM 1
Continuous presence of a registered nurse	A registered nurse is not always present during the day shift in the living unit.	PresenceRN 0
	A registered nurse is always present during day shift in the living unit.	PresenceRN 1
Special qualification of head nurse in psychogeriatric care	The head nurse of the living unit has no special qualification in psychogeriatric care.	Jobqual 0
	The head nurse of the living unit has a special qualification in psychogeriatric care.	Jobqual 1
Additional financing regulated by a special agreement	Living unit is not additionally financed.	Finance 0
	Living unit is additionally financed.	Finance 1
Living concept	Integration (residents with and without dementia live together in one living unit).	Segregative 0
	Segregation (residents with dementia live together in one living unit).	Segregative 1
Residents-per-registered nurse ratio (defined as nurses with a minimum education of three years).	The RNRatio is greater than the median (cut-off: median = 18).	RNRatio 0
	The RNRatio is less than or equal to the median (cut-off: median = 18).	RNRatio 1
Certified nursing assistant ratio (defined as nurses with a minimum education of one year)	There are no Certified nursing assistants working on the living unit.	CNARatio 0
	There are Certified nursing assistants working on the living unit.	CNARatio 1
Residents-per- nursing assistant ratio (defined as nurses without any education)	The NARatio is greater than the median (cut-off: median = 16).	NARatio 0
	The NARatio is less than or equal to the median (cut-off: median = 16).	NARatio 1
Residents-per-service staff member ratio	The SSMRatio is greater than the median (cut-off: median = 28).	SSMRatio 0
	The SSMRatio is less than or equal to the median (cut-off: median = 28).	SSMRatio 1
Accessible outdoor area	There is no accessible outdoor area.	Outdoor 0
	The residents can go out alone.	Outdoor 1
	The residents can only go out in the presence of a caregiver.	Outdoor 2

The variables were developed based on theoretical knowledge about the field of German nursing homes derived from the literature on their regulation. Some of the questionnaire items were taken from the official quality audits that are performed regularly by the Medical Review Board of the Statutory Health Insurance Funds. Other items were newly developed for the study. The questionnaire was evaluated for content validity by experts in the field and future users using expert interviews and a multimethod pretest. The development of the care unit questionnaire followed the same methodological procedure as the DemCare-Q questionnaire [21]. The reliability has not been validated yet. In addition to the structural characteristics, resident variables were included to determine the age, sex, presence of dementia diagnosis and severity of dementia [22]. These variables were used exclusively to further describe the identified clusters and did not contribute to their calculation.

### Statistical analysis

Multiple correspondence analysis (MCA) and agglomerative hierarchical cluster analysis (AHC) were used to develop the typology of living units. First, an MCA was used whose principal components represent synthetic quantitative variables that summarize all categorical variables [23]. This is a dimension-reducing procedure that selects a few characteristic combinations from the many possible characteristics so that as much information as possible is retained from the data. Second, an AHC is performed with the dimensionally reduced data; this method is suitable for identifying groups of living units that are mapped in the geometric structures of the MCA [24]. The statistical software R was used to conduct the statistical analyses [25]. MCA and AHC analyses were performed with the R package “FactoMineR” using the MCA and HCPC functions [26]. The plots of the results were generated using the R Package “factoextra” [27]. The R-code and the raw data for the living units are available in the supplemental information. Finally, the residents’ data are compared between the clusters by using the R package “atable” [28]. This table can be retrieved in the supplementary information.

To make the procedure transparent and the graphical results comprehensible, the following sections contain a brief description of the methods used. This includes the explanation of methodical analysis steps that provide a basis for decision-making regarding the presentation of results. We decided how much information is retained by the MCA and how many clusters are formed by the AHC.

### Correspondence analysis

Correspondence analysis (CA) is a descriptive data analysis technique that enables the graphical representation of both the row and column characteristics of a contingency table in the same low-dimensional spatial area. Thus, CA belongs to a family of methods (factor analysis and principal component analysis) that reveal patterns in complex datasets. MCA is a specific application of CA that can be understood as a generalization of CA to cases in which there are more than two variables [29]. Therefore, we apply the MCA to a complete disjunctive table [30] with living units in the rows and variables (structural characteristics) in the columns. The deviation of these row or column profiles from their respective average profile, which is displayed by the centroid of the graphical data representation in the map, is used as a measure of the variance in the data. This measure of variance is called inertia in the context of MCA.

In a nutshell, MCA calculates the singular value decomposition (SVD) of the complete disjunctive table, yielding a set of eigenvalues *λ*_*s*_ and corresponding eigenvectors (here called axes) [31]. The eigenvalues are also called inertia in the context of MCA. The researcher has to choose how many of the axes and eigenvalues he or she wants to omit to reduce the dimensions of the data cloud. Here, the inertia provides guidance. To determine the number of axes (dimensions) to be analyzed, various information about the percentage of explained inertia and the interpretability of each axis is taken into account. For high-dimensional data sets, such as those presented in this article, the modified inertia rates should also be considered because the inertia rates of the first dimension are usually low. The modified inertia rates highlight the significance of the first principal axis [32]. This information is used to calculate the best low-dimensional solution capable of distinguishing geometric patterns in the data by mapping each category of the structural characteristics and each living unit as a point in the same Euclidean space [33]. Consequently, we use a multiple correspondence analysis to visualize the relevant relationships between the structural characteristics by their distances from each other. Thus, all variables are included in the statistical model, but the complexity of their representation in the MCA-map is reduced by the dimensional reduction of the axes. In addition, the MCA provides information about the distances between the living units, which we used in the second step to identify types of similar living units by performing the AHC method.

### Agglomerative hierarchical clustering on principal components

Following the MCA, an AHC was performed, clustering the living units on the basis of the calculated principal components of the MCA [29]. This implies that the living units are clustered by using their coordinates on the principal axes, i.e. their Euclidean distance from one another. Consequently, the agglomerative procedures start the calculation process at the “finest partition”, which means that each living unit initially represents a cluster. The further calculation process merges two clusters and is continued step by step until all living units are united into a single cluster. This creates a hierarchical relationship between the clusters of the living units, which can be visually represented by a fixed order of the cluster solutions in the dendrogram (see Fig. 2). The Ward process [34] applied here is of particular importance among the agglomerative processes. The purpose is to merge the living units (clusters of living units) that increase the inertia in a cluster as little as possible. The total inertia consists of the “within-cluster inertia”, which describes the deviations of the living units (points) from their cluster center, and the “between-clusters inertia”, which describes the deviations between the individual cluster centers and the overall center of all living units. An analysis of the inertia decomposition is valuable to describe the quality of the cluster solution. The aim is to identify an appropriate cluster solution that minimizes the variability of the “within-cluster” or maximizes the “between clusters” variability. For the combined application of MCA and AHC, two principles of conduct have been taken into account that are recommended in the method literature [29]:

1. The extracted dimensions of the MCA, which represent very insignificant proportions of explained inertia, can be interpreted as statistical “noise”. It is therefore recommended for the subsequent performance of the AHC that only those axes are included in the analyses that explain a high proportion of total inertia (approx. 80 to 90% in total).
2. The axes retained in the MCA should be interpretable. As a rule, this makes the results of the AHC easier to interpret.

Subsequent to the hierarchical cluster analysis, a test value can be applied to check the extent to which the categories correspond with the identified clusters. The v-test is a test to compare the proportion of the category in a cluster compared to the proportion of the category in the global dataset. The test is used to identify significant categories (*p* < 0.05) that define the clusters [35].

## Results

The data of the 103 living units applied to calculate the MCA and AHC include *Q* = 19 variables with a total of *K* = 39 categories. The frequency distributions of the categories are displayed in Table 2.

**Table 2:** Absolute and relative frequencies of categories

Variable category (Shortname)	% (*N* = 103)
Number of beds in living unit ≤15 (Size 0)	23% (24)
Number of beds in living unit > 15 (Size 1)	77% (79)
Living units do not exclusively have single rooms (SRoom 0)	70% (72)
Living units have only single rooms (SRoom 1)	30% (31)
Living unit was not specially built for people with dementia (Build 0)	54% (56)
Living unit was built specially for people with dementia (Build 1)	46% (47)
Living unit is not located in a separate building (Separate 0)	31% (32)
Living unit is located in a separate building (Separate 1)	69% (71)
Living unit is not protected by an exit control (Guarded 0)	83% (86)
Living unit is protected by exit controls (Guarded 1)	17% (17)
Furnishings are solely functional (Furniture 0)	13% (13)
Furnishings are functional and individual (Furniture 1)	87% (90)
Lunch is not cooked in the kitchen of the living unit (Selfcook 0)	73% (75)
Lunch is cooked in the kitchen of the unit (Selfcook 1)	27% (28)
All meals are not served homestyle on the table (Mealserv 0)	80% (82)
All meals are served home style on the table (Mealserv 1)	20% (21)
Nurses do not work exclusively in one unit (AssignN 0)	6.8% (7)
Nurses work exclusively in one living unit (AssignN 1)	93% (96)
Service workers do not work exclusively in one living unit (AssignSSM 0)	25% (26)
Service workers work exclusively in one living unit (AssignSSM 1)	75% (77)
A registered nurse is not always present during the day shift (PresenceRN 0)	9.7% (10)
A registered nurse is always present during day shift (PresenceRN 1)	90% (93)
No special qualification in psychogeriatric care (Jobqual 0)	75% (77)
Special qualification in psychogeriatric care (Jobqual 1)	25% (26)
Living unit is not additionally financed by a special agreement (Finance 0)	84% (87)
Living unit is additionally financed by a special agreement (Finance 1)	16% (16)
Integrative living concept (Segregative 0)	61% (63)
Segregated living concept (Segregative 1)	39% (40)
Residents-per-registered nurse ratio is greater than the median (RNRatio 0)	49% (50)
Residents-per-registered nurse ratio is less than or equal to the median (RNRatio 1)	51% (53)
There are no Certified nursing assistants working on the living unit (CNARatio 0)	70% (72)
There are Certified nursing assistants working on the living unit (CNARatio 1)	30% (31)
Residents-per- nursing assistant ratio is greater than the median (NARatio 0)	50% (51)
Residents-per- nursing assistant ratio is less than or equal to the median (NARatio 1)	50% (52)
Residents-per-service staff member ratio is greater than the median (SSMRatio 0)	49% (50)
Residents-per-service staff member ratio is less than or equal to the median (SSMRatio 1)	51% (53)
There is no accessible outdoor area (Outdoor 0)	6.8% (7)
The residents can go out alone (Outdoor 1)	80% (82)
The residents can only go out in the presence of a caregiver (Outdoor 2)	14% (14)

### Relations between the characteristics and the living units: results of the MCA

The calculation of the total inertia of the data amounts to *K*/*Q* − 1 = 1.053 and is distributed over a total of *K* − *Q* = 20 eigenvalues. The average eigenvalue is $\overline{\lambda}=1/Q=0.052$ and explains 4.93% of the total inertia.

Table 3 illustrates the proportion of explained inertia for each axis in decreasing order and thus provides the information needed to make decisions about the number of axes to be included in the analysis. The second axis brings the cumulated modified inertia rate to 90.90%. Therefore, only the first two axes will be interpreted in the results of the MCA.

**Table 3:** Inertia of axes, inertia rates, and modified rates

Axes	Inertia %	Cumulative inertia %	Modified inertia %	Modified cumulative inertia %
1	17.44	17.44	60.76	60.76
**2**	**13.76**	**31.21**	**30.13**	**90.90**
3	8.15	39.36	3.90	94.80
4	7.38	46.73	2.22	97.01
5	7.36	54.09	2.19	99.20
6	6.36	60.45	0.73	99.93
7	5.35	65.80	0.05	99.97
8	5.26	71.06	0.03	100.00
9	4.85	75.92	0.00	100.00
10	4.13	80.05	0.00	100.00
**11**	**3.66**	**83.71**	**0.00**	**100.00**
12	3.00	86.71	0.00	100.00
13	2.32	89.03	0.00	100.00
14	2.12	91.15	0.00	100.00
15	1.98	93.13	0.00	100.00
16	1.82	94.95	0.00	100.00
17	1.61	96.56	0.00	100.00
18	1.53	98.09	0.00	100.00
19	1.10	99.19	0.00	100.00
20	0.81	100.00	0.00	100.00

**Fig. 1:**
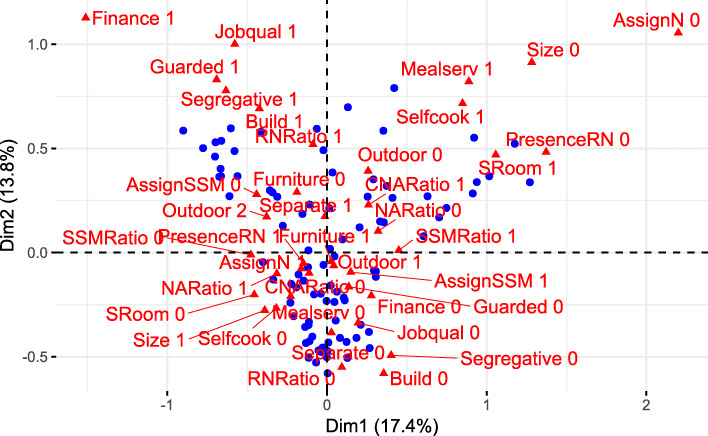
MCA map for the superimposed representation of living units (blue points) and structural characteristics (red triangles)

The first axis *λ*_1_ explains 17.44% of the total inertia, and the second axis *λ*_2_ explains 13.76% of the total inertia. Thus, the MCA map (Fig. 1) represents 31.21% of the total inertia. For the interpretation of the principal axes, the categories that contribute significantly to the explanation of the principal axis are informative. These include all categories whose contribution exceeds the average contribution of 2.56%.

The first principal axis applies to the following categories: “living unit has a size ≤ 15 beds” (Size 0), “living unit is additionally financed” (Finance 1), “living unit has only single rooms” (SRoom 1), “nurses do not work exclusively in one unit” (AssignN 0), “lunch is cooked in the kitchen of the unit” (Selfcook 1), “a registered nurse is not always present” (PresenceRN 0), “all meals are served homestyle on the table” (Mealserv 1), “segregated living concept” (Segregative 1), “do not exclusively have single rooms” (SRoom 0), “living unit has a size > 15” (Size 1), “residents-per-service staff member ratio is less than or equal to the median” (SSMRatio 1), “residents-per-service staff member ratio is greater than the median” (SSMRatio 0), and “integrative living concept” (Segregative 0). The categories are sorted according to their contributions, so that the first category Size 0 explains the main contribution to the first axis. A substantial contribution to the second principal axis is made by the following categories: “no special qualification in psychogeriatric care” (Jobqual 0), “segregated living concept” (Segregative 1), “built specially for people with dementia” (Build 1), “is additionally financed” (Finance 1), and “living unit has a size ≤ 15” (Size 0). These categories each explain between seven and 10 % of the second principal axis.

The categories that are close to each other, such as “living unit is additionally financed” (Finance 1), “special qualification in psychogeriatric care” (Jobqual 1), “living unit is protected by exit controls” (Guarded 1), etc. are correlated positively with each other and describe the corresponding living units in this area.

Binary categories always correlate negatively and are located opposite to each other. Most of the living units that are distinguished by the binary categories are scattered in the left and right upper areas of Figure1. These living units differ significantly from the living units displayed on the second principle axis below the centroid.

### Identifying the types of living units: results of the AHC

**Fig. 2:**
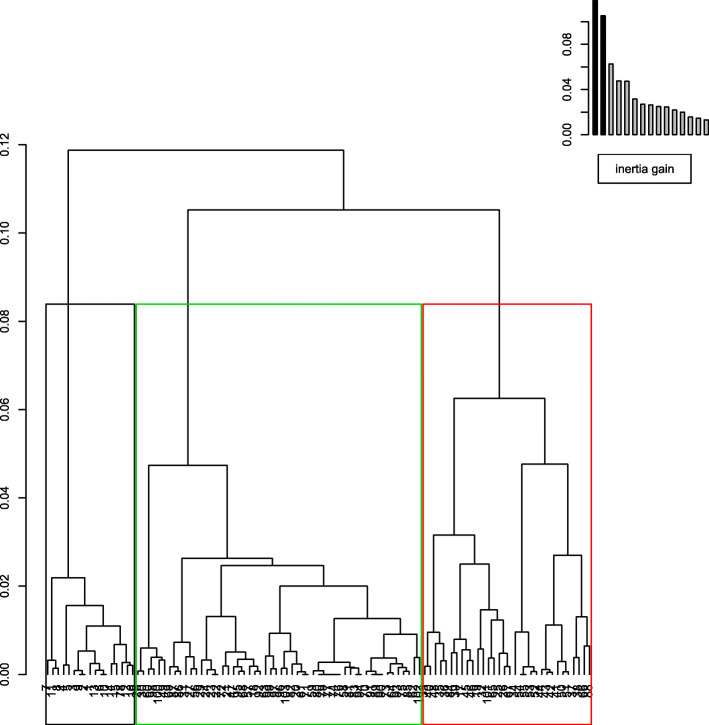
Dendrogram for the hierarchical representation of the living unit clusters

The numbers of clusters with the highest percentage decrease in the gain of the between-clusters inertia are marked by a bend (elbow criterion) in the curve of the inertia gain in Fig. 2. For this reason, three clusters were chosen. The proportion of “between-clusters inertia” that can be measured is 25.41% for a three-cluster solution.

In our analysis we choose 11 axes to calculate the AHC that summarize 83.71% of the total inertia.

**Fig. 3:**
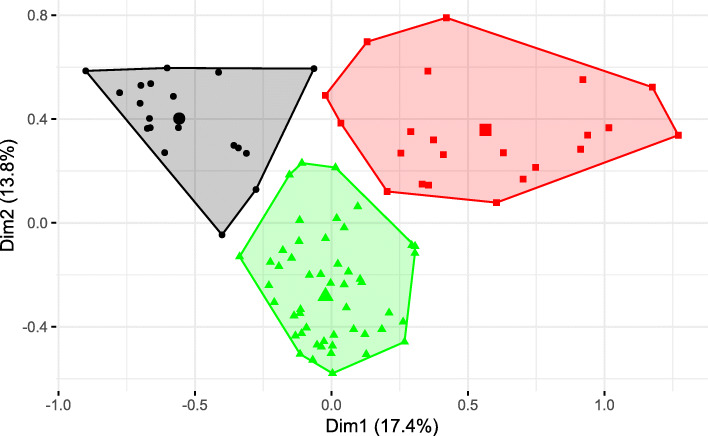
MCA map with clusters (black = dementia special care units, green = usual care, red = house community)

Figure 3 displays the convex hulls of the three cluster solutions in the correspondence space of the MCA map. The two clusters in the upper left (living units = circles) and upper right area (living units = squares) differ in the first dimension. These clusters are related to the categories that make a significant contribution to the first principal axis. The largest cluster (living units = triangles) is close to the centroid and differs in the second principle axis. This cluster represents the average living unit type and is associated with the categories that contribute significantly to the second principal axis.

By applying the v-test, the structural characteristics that clustered the respective living units were determined. The test results show that each of the three clusters in Fig. 3 occurs with a specific combination of categories. Table 4 illustrates these combinations, which leads us to the content-related definition of our three cluster types. We designate the three clusters as “house community”, “dementia special care units” and “usual care”.

**Table 4:** Clusters and their characteristic categories

Dementia special care units (*N* = 21)	Usual care (*N* = 59)	House community (*N* = 23)	Not significant
Finance 1 (100, 76, 16%)	Size 1 (73, 98, 77%)	Size 0 (92, 96, 23%)	Separate 1
Jobqual 1 (65, 81, 25%)	Jobqual 0 (73, 95, 75%)	SRoom 1 (68, 91, 30%)	Guarded 0
Segregative 1 (50, 95, 39%)	Finance 0 (68, 100, 84%)	Selfcook 1 (57, 70, 27%)	Furniture 0
Build 1 (43, 95, 46%)	Build 0 (80, 76, 54%)	AssignN 0 (100, 30, 7%)	Furniture 1
RNRatio 1 (34, 86, 51%)	Segregative 0 (76, 81, 61%)	PresenceRN 0 (80, 35, 10%)	Outdoor 1
Guarded 1 (53, 43, 17%)	RNRatio 0 (80, 68, 49%)	Mealserv 1 (57, 52, 20%)	Outdoor 2
SRoom 0 (28, 95, 70%)	SRoom 0 (69, 85, 70%)	SSMRatio 1 (36, 83, 51%)	AssignSSM 0
SSMRatio 0 (32, 76, 49%)	AssignN 1 (61, 100, 93%)	Finance 0 (26, 100, 84%)	AssignSSM 1
Size 1 (25, 95, 77%)	Selfcook 0 (65, 83, 73%)	Outdoor 0 (57, 17, 7%)	CNARatio 1
Selfcook 0 (25, 90, 73%)	Separate 0 (75, 41, 31%)		CNARatio 0
	Mealserv 0 (63, 88, 80%)		NARatio 1
	PresenceRN 1 (61, 97, 90%)		NARatio 0

Legend:

The percentages in brackets (*p*_1_, *p*_2_, *p*_3_) that determine the statistical significance (*p*-value) of the category by applying the v-test are defined as follows:

*p*_1_ specifies the percentage of living units possessing the corresponding category that are in the respective cluster. For example, all 16 living units with the characteristic Finance 1 are included in the cluster “Dementia special care units”, i.e., $100\%=100\ast \frac{16}{16}$.

*p*_2_ specifies the percentage of living units in the cluster possessing the corresponding category. For example, 16 out of 21 total living units in the cluster “Dementia special care units” have the characteristic Finance 1, i.e., $76\%\approx 100\ast \frac{16}{21}$.

*p*_3_ specifies the percentage of living units possessing the corresponding category in the sample

For example, 16 out of 103 total living units have the characteristic Finance 1, i.e., $16\%\approx 100\ast \frac{16}{103}$.

The categories in Table 4 describing the clusters are sorted in decreasing order according to their significance such that the first categories have the lowest *p*-values as a result of the v-test. The categories (Finance 1, Size 1 and Size 0) that are most significant for their respective clusters are therefore at the head of the table. The v-test and p-values can be calculated using the R-code in the supplementary information. To present a summarized table as a result, we have decided to provide the percentages in the brackets only, as they are more descriptive for the distribution of a category. All listed categories used to describe the three clusters satisfy the *p* <  0.05 requirement. With the exception of the last column, “Not significant”, all categories that do not provide significant information for the clusters are displayed.

Furthermore, we observe three different cases of attributions in the categories of the clusters in Table 4. Some examples are as follows: The first case of attribution concerns categories that are only informative for a particular cluster. We describe this case as a “unique characteristic”. This applies, for example, to the “living unit is protected by exit controls” (Guarded 1) category in the “dementia special care units” cluster. The category “Guarded 1” is only significant in Cluster “dementia special care units” and not in other clusters. Therefore, we call this category “unique characteristic”. The second case concerns dichotomous categories relating to different clusters. We define this case as “strong difference”. This is valid for the categories “living unit was not specially built for people with dementia” (Build 0) and “living unit was built specially for people with dementia” (Build 1) because Build 1 relates to the cluster “dementia special care unit” and Build 0 to the cluster “usual care”. The third case will be applicable when a category is related to two or more clusters. We define this case as “intersection”. This applies to the category “do not exclusively have single rooms” (SRoom 0), which is indicative of both the cluster “dementia special care unit” and the cluster “usual care”. However, it should be noted that the second case also applies to the category “living units do not exclusively have single rooms” (SRoom 0) because “living units have only single rooms” (SRoom 1) is informative for the cluster “house community”. Categories describing the second case are particularly suitable for describing differences between two clusters.

Table 4 shows that these category combinations allow clear distinctions to be made from the cluster “usual care”. The five top categories of the cluster “dementia special care unit” and cluster “house community” can be distinguished by the dichotomous categories of the cluster “usual care”.

In contrast, the differences between the clusters “dementia special care units” and “house community” are distinguished more by their unique characteristics. This distinction is exemplified by the fact that categories such as “special qualification in psychogeriatric care” (Jobqual 1), “segregated living concept” (Segregative 1) and “living unit is protected by exit controls” (Guarded 1) are informative for the cluster “dementia special care units”, but, including their dichotomous category, have no significance for the cluster “house community”.

To further validate the identified types, we additionally conducted some descriptive analysis to describe differences between the residents who are living in the living units. The examination of the resident data shows no differences regarding the variable “sex”. However, there are clear differences in the “age”, “diagnosis of dementia” and “severity of dementia”. The relative frequencies of dementia diagnosis and severe dementia are significantly higher in the “dementia special care units” cluster. These results (resident characteristics of the three clusters of dementia special care units, usual care, and house community) are presented as a table in the supplementary information.

### Comparison between the a priori defined types and the types identified by the explorative clustering technique: results of the cross table

If we compare the current types identified with the explorative clustering method to the a priori defined types, we can see that the different types of development techniques had an impact on the affiliation of the 103 living units to the types. To illustrate this, Table 5 presents a cross-table that contrasts the affiliations of the living units with the different types.

**Table 5:** Cross table for comparison between the a priori defined types and the types identified by the explorative clustering technique

	Dementia special care units	Usual care	House community
LSLUI	3	11	1
LSLUII	16	0	0
LILU	1	47	0
SSLU	1	0	8
SILU	0	1	14

Legend:

LSLU I-Large segregated living units without extra funding

LSLU II-Large segregated living units with extra funding

LILU-Large integrated living units without extra funding

SSLU-Small segregated living units without extra funding

SILU-Small integrated living units without extra funding

One can see that all of the living units that were formerly affiliated with the type “large segregated living units with additional financing regulated by an agreement” (LSLU II) are now affiliated with the type “dementia special care units”. However, three living units that were formerly affiliated with the type “large segregated living units without extra funding” (LSLU I) are also affiliated with the type “dementia special care units”. It is surprising that one living unit that was formerly affiliated with the type “large integrated living units without extra funding” (LILU) is now also affiliated with “dementia special care units”. This may be explained by the fact that this living unit does not have the characteristic “segregative living concept” (Segregative 1) but is defined by the type specific characteristics “built specially for people with dementia” (Build 1), “special qualification in psychogeriatric care” (Jobqual 1), "residents-per-registered nurse ratio is less than or equal to the median" (RNRatio 1), “do not exclusively have single rooms” (SRoom 0), “residents-per-service staff member ratio is greater than the median”(SSMRatio 0), “lunch is not cooked in the kitchen of the living unit” (Selfcook 0) and “large size” (Size 1).

When looking at the type “usual care”, it is clear that the majority (47 of 59) were formerly affiliated with the type “large integrated living units without extra funding” (LILU). However, 11 living units from the type “large segregated living units without extra funding” (LSLU I) are now affiliated with the “usual care” type. The type “house community” is more or less consistently compounded by living units that were formerly affiliated with the small living units (integrated and segregated without extra funding).

Again, what is surprising is that one living unit that was formerly affiliated with the type “large segregated living units without extra funding” (LSLU I) is now affiliated with the type “house community”. This can be explained by the categories “lunch is cooked in the kitchen of the living unit” (Selfcook 1), “all meals are served home style on the table” (Mealserv 1), “residents-per-service staff member ratio is less than or equal to the median” (SSMRatio 1) and “living unit is not additionally financed” (Finance 0), which were evident in this living unit.

## Discussion

The aim of this study was to empirically develop a typology of living units based on their structural characteristics. Using an explorative clustering technique on data from 103 living units in 51 nursing homes, we identified three different clusters (types). We designated the types as “house community”, “dementia special care units” and “usual care”. The three categories that have the greatest influence on the formation of these types are named below.

The categories that showed the strongest influence on the first type, “dementia special care units,” were “additionally financed” (Finance 1), “special qualification in psychogeriatric care” (Jobqual 1) and “segregated living concept” (Segregative 1). The categories that contributed most to the second type, “usual care,” were “large size” (Size 1), “no special qualification in psychogeriatric care” (Jobqual 0) and “not additionally financed” (Finance 0). The categories that showed the strongest influence on the third type, “house community,” were “small size” (Size 0), “living unit with only single rooms” (Sroom 1) and “cooked lunch in the kitchen of the living unit” (Selfcook 1). Prior to this study, we used a deductive approach to define living unit types and used the variables size, living concept, and finance (Palm et al. [19]).

If we compare the types identified with the empirical exploratory methods to these a priori defined types, we can see that some categories that were used for definition also have a strong impact on the types developed in the empirical MCA model, whereas others have not. Two types were defined using the categories “large size” (Size 1), “segregative living concept” (Segregative 1) and the variable “additional financing regulated by a special agreement” (Finance 0 and Finance 1). Hence, they differed with respect to the additional financing variable, which was present in one type but not in the other. In the MCA model, the categories “no additional financing regulated by a special agreement” (Finance 0) and “large size” (Size 1) correlate with each other, but there is no correlation between the categories “segregative living concept” (Segregative 1) and “ large size ” (Size 1). However, the category “segregative living concept” (Segregative 1) correlates strongly with the category “additional financing regulated by a special agreement” (Finance 1) but not with “large size” (Size 1). The categories “small size” (Size 0) and “segregated living concept” (Segregative 1) that were also used to define the type “small segregated living units without extra funding” (SSLU) a priori showed no correlation in the MCA model.

If we look at the variables that were significant in determining the empirically developed types, it becomes apparent that other variables play roles that were not considered in the a priori definition. This observation applies to “building specific for residents with dementia”, “special qualification of the head nurse in psychogeriatric care”, “availability of single rooms”, “resident-per-service staff member ratio (is less or equal than the mean)”, “possibilities to cook lunch in the living unit”, etc.

In the present study, we also showed which variables and categories do not contribute to the empirical cluster model “constant assignment of service staff” (AssignSSM 0 and AssignSSM 1), “certified nursing assistant ratio” (CNARatio 0 and CNARatio 1), “residents-per-nursing assistant ratio” (NARatio 0 and NARatio 1), “furnishing of public rooms” (Furniture 0 and Furniture 1), “living unit is located in a separate building” (Separate 1), “living unit is not protected by an exit control” (Guarded 0). Some of these variables (“furnishing of public rooms” and “constant assignment of service staff”) also did not show significant differences between the a priori defined five living unit types. In contrast to the previous results, “intersections”, “unique characteristics” and “strong” “differences” between the clusters can be identified for the empirical cluster solution.

This is evident in the classification of the categories that are described for the results of Table 4. These attribution possibilities result from the multivariate static model, enabling the relationships between the clusters to be described in detail. Findings of this kind cannot be obtained from the a priori defined types, since they are derived from the correlations to the characteristics between the empirical types. This can be seen as an additional benefit of the empirical approach chosen in the current study.

Furthermore, the cluster association in the current results is not determined by the fact that the living units have all the cluster-specific characteristics in Table 4. Rather, the probability that a living unit belongs to a particular cluster increases with the presence of each additional cluster-specific characteristic. Thus, in terms of the data, it is probable (92% chance) that a living unit with the characteristic “small size” (Size 0) belongs to the cluster “house community”.

The probability increases to 95% if the characteristic “lunch is cooked in the kitchen of the living unit” (Selfcook 1) is specified in addition to the characteristic “small size” (Size 0). When a living unit has the first three characteristics of the cluster “house community”, the affiliation is 100%. This offers the advantage that probabilities of affiliation can be calculated for all existing combinations of characteristics in the living units data set. In contrast to the a priori defined types, differences in the affiliation to the empirical types of single living units can be defined. From a methodological perspective, it should be noted that the formation of a typology of living units based on complex characteristic correlations can be appropriately described using a multivariate statistical method. A methodological approach such as the one applied in the present study is suitable for mapping the multiple interrelationships of structural characteristics in organizational health and thus represents a powerful tool for describing the care landscape [33, 36]. An advantage of this explorative analysis is that it delivers a cluster solution that fits the data. In the previously published results, eight possible types were defined a priori, of which only five types could be achieved in the data [19].

Finally, a comparison of the “dementia special care units” cluster in Table 4 with more recent research shows that empirical studies that investigate the influence of a Dementia Special Care Unit on residents’ outcomes often do not use multiple indicators to define them but rely on single indicators such as the availability of specially trained staff [37], SCU placement variable of the MDS 2.0 [38] or the US OSCAR reporting system [39, 40]. Other studies combine several indicators based on an a priori set definition [41]. The latter used the indicators specially trained staff, 100% of the residents of the unit have a dementia and the unit is closed. However, in our sample of living units, these combinations of indicators are applicable only to living units with additional funding regulated by an agreement, not to all living units that exclusively house residents with dementia.

### Limitations

There are methodological limitations of the DemenzMonitor study and the present study that limit the external validity of the results. The participating institutions are spread over 11 federal states. It should be noted here that the distribution of institutions among the federal states in the data set does not correspond to the actual distribution of inpatient geriatric care institutions in Germany. Therefore, the results cannot be considered representative of German care institutions in general. A further methodological limitation relates to the dichotomization of variables. For the staffing variables, the ratio was split using the median. Such a definition is difficult to justify and is normative. This causes information to be lost. An alternative would be to use methods that can map both categorical and metric variables in a model. Pagès recommends more advanced methods, such as Factorial Analysis of Mixed Data [30].

## Conclusions

We identified systematic differences based on a large number of criteria. These results lead to a complex type formation, as seen from the fact that the types are described by nine or more characteristics. This supports the assumption that definitions that are solely based on size or living concept ignore the diversity within these groups [19].

A main result of the comparison is that the five a priori types would not be formed in the multivariate model because there are major groups of characteristics that correspond more to each other than to other characteristics and thus lead to a more stable cluster solution. If the intersections of the five cluster solutions and the three cluster solutions are considered, it becomes apparent that the variable “additional financing regulated by a special agreement” and “size of the living unit” are particularly suitable for distinguishing between them. The variable “living concept” has a significantly lower impact on the differentiation of clusters. This can be seen, on the one hand, in the ranking of the categories and, on the other hand, in the result that the variable is insignificant for the cluster “house community”.

Regarding a classification of living units based on the present study, the following practical recommendation can be made: It can be assumed that a living unit belongs to a cluster if it has three or more of the characteristics shown in Table 4. If we look at the first three characteristics of the clusters, we see the following allocation probability:

If a living unit is assigned the characteristics “additional financing regulated by a special agreement” (Finance 1), “special qualification in psychogeriatric care” (Jobqual 1) and “segregative living concept” (Segregative 1), then it is 100% in the cluster “Dementia special care units”.
If a living unit has the characteristics “living unit has a size > 15” (Size 1), “no special qualification in psychogeriatric care” (Jobqual 0) and “not additionally financed” (Finance 0), then it is 96.49% in the cluster “Usual care”.
3. If a living unit has the characteristics “living unit has a size ≤ 15 beds” (Size 0), “living unit has only single rooms” (SRoom 1) and “cooked lunch in the kitchen of the living unit” (Selfcook 1), then it is 100% in the cluster “House community”.

If the characteristics in the ranking of the table are higher, the classification of the corresponding living unit is more reliable.

Once confirmed, the typology can also provide important information for intervention and implementation studies, where the context of nursing homes needs to be explained thoroughly to evaluate transferability. If nursing home care units that participate in an intervention or implementation trial are assigned to one of the identified types, the results from different studies become more comparable. At the same time, it may be possible to investigate whether an intervention shows better effects in one type than in others. This information is again important for evaluating the transferability of intervention effects in practice.

The implications relevant to future organizational nursing and care research can be summarized as follows: Because the study is designed as an explorative study, no power analysis was performed to identify and validate specific clusters. Therefore, it would be desirable for future studies to test the three-cluster solution on a more representative sample with the use of confirmatory techniques. Furthermore, it is still necessary to answer the question of whether we can determine differences in the quality of care and residents’ outcomes between dementia-specific and traditional care units. To this end, it remains important to represent the existing differences among the living units, which result from the complex diversity of specialized institutions, as accurately as possible in a typology.

## Supplementary information

**Additional file 1: Table.** Resident characteristics of the three clusters of dementia special care units, usual care and house community.

**Additional file 2.** Code and raw data

## Data Availability

The data of the living units and the R code for calculating the typology of living units are available in the supplementary information. Data of the residents are not provided for data protection reasons.

## Abbreviations

AHC: Agglomerative Hierarchical Cluster Analysis
CA: Correspondence Analysis
DSCUs: Dementia Special Care Units
LILU: Large integrated living units without extra funding
LSLU I: Large segregated living units without extra funding
LSLU II: Large segregated living units with extra funding
MDS: Minimum Data Set
MCA: Multiple Correspondence Analysis
OSCAR: Online Survey, Certification, and Reporting
SCU: Special Care Unit
SILU: Small integrated living units without extra funding
SSLU: Small segregated living units without extra funding
SVD: Singular value decomposition
