# Supplementary material for: Empirical development of a typology on residential long-term care units in Germany - results of an exploratory multivariate data analysis
Source: BMC Health Serv Res. 2020 Jul 11;20:646. doi: 10.1186/s12913-020-05401-4 (PMC7353672; doi:10.1186/s12913-020-05401-4)
Supplement: Supplementary file 1 — Additional file 1: Table. Resident characteristics of the three clusters of dementia special care units, usual care and house community. [file 12913_2020_5401_MOESM1_ESM.docx]

| Cluster | Dementia special care units | Usual Care | House community | p |
| --- | --- | --- | --- | --- |
| Observations | 24 | 1235 | 247 |  |
| Age |  |  |  |  |
| Mean (SD) | 81 (9) | 83 (8.9) | 84 (8.7) | 0.0011 |
| valid (missing) | 324 (0) | 1235 (0) | 247 (0) |  |
| Sex |  |  |  |  |
| female | 75% (243) | 77% (953) | 73% (180) | 0.3 |
| male | 25% (81) | 23% (282) | 27% (67) |  |
| missing | 0% (0) | 0% (0) | 0% (0) |  |
| Diagnosis of dementia |  |  |  |  |
| no | 2.2% (7) | 33% (407) | 23% (56) | <0.001 |
| yes | 98% (317) | 67% (822) | 76% (188) |  |
| missing | 0% (0) | 0.49% (6) | 1.2% (3) |  |
| DSS |  |  |  |  |
| No dementia (0-2) | 1.5% (5) | 25% (306) | 21% (51) | <0.001 |
| Mild-moderate dementia (3-7) | 13% (42) | 31% (388) | 30% (73) |  |
| Severe dementia (8-14) | 85% (277) | 44% (538) | 50% (123) |  |
| missing | 0% (0) | 0.24% (3) | 0% (0) |  |

Table: Resident characteristics of the three clusters of dementia special care units, usual care and house community.
